# Canine-Assisted Therapy and Quality of Life in People With Alzheimer-Type Dementia: Pilot Study

**DOI:** 10.3389/fpsyg.2019.01332

**Published:** 2019-06-06

**Authors:** Leticia Sánchez-Valdeón, Elena Fernández-Martínez, Sara Loma-Ramos, Ana Isabel López-Alonso, Enrique Bayón Darkistade, Valentina Ladera

**Affiliations:** ^1^SALBIS Research Group, Faculty of Health Sciences, University of León, León, Spain; ^2^Department of Nursing and Physiotherapy, University of León, León, Spain; ^3^Faculty of Psychology, University of Salamanca, Salamanca, Spain

**Keywords:** quality of life, benefits, Alzheimer’s, canine-assisted therapy, severe cognitive decline, person-centered care

## Abstract

**Background:**

With the aim of improving the quality of life of people with Alzheimer’s disease, primarily as regards behavioral and psychological symptoms, we implemented canine-assisted therapy in a group of people with this disease.

**Methods:**

We conducted a quasi-experimental study, with a simple pre-post case series design. Participants comprised 10 Alzheimer’s day care center users presenting severe or very severe cognitive decline. The measurement instrument employed was the Quality of Life in Late-Stage Dementia (QUALID) scale.

**Results:**

By the end of the therapy, 100% of participants showed an improvement in physical, behavioral, and psychological aspects. The total scores of the QUALID scale for the three different evaluation times (before the therapy and 6 and 12 months after starting) after the canine-assisted therapy intervention were smaller and less dispersed. The total score for the QUALID scale decreased significantly (*p* < 0.05) at 6 and 12 months after starting therapy. An analysis by item revealed that the scores of all of them fell during the course of the therapy.

**Discussion:**

Our study provides evidence of the significant benefits of canine-assisted therapy for quality of life in people with Alzheimer’s disease.

## Introduction

According to the World Health Organization (WHO), dementia affects approximately 47.5 million people in the world, and 7.7 million new cases are diagnosed every year (World Health Organization, 2018). In the period 1990–2008, around 600,000 people in Spain were diagnosed with dementia, of whom 400,000 were diagnosed with Alzheimer’s disease (De Pedro-Cuesta et al., 2009), exerting a significant impact on families, costs, and health and welfare services.

As indicated by the Director-General of the WHO, Tedros Adhanom Ghebreyesus, the prevalence of dementia is an alarming problem throughout the world, and as a result, “*we must pay more attention to this growing challenge and ensure that all people living with dementia, wherever they live, receive the care they need.*” The goal of the Global Action Plan on the public health response to dementia 2017–2025 (World Health Organization, 2018) is to improve the lives of people with dementia, their careers, and families, while decreasing the impact of dementia on communities and countries, which represents a major challenge due to worldwide population aging.

Alzheimer’s disease is defined clinically by causing dementia and corresponds to 50–60% of all dementias, being the most prevalent (Alameda et al., 2012; Claassen, 2015). No curative treatment is currently available and the disease generates high healthcare, social, and family costs (Leon-Salas and Martínez-Martín, 2010). Correct diagnosis permits identification of the treatable causes of dementia, where these exist, or the possibility of slowing the process in other cases (American Psichiatric Association, 2002; Costa and Castiñeira, 2016). It is essential to establish a suitable therapeutic plan (pharmacological and non-pharmacological) in order to maintain independent functioning for as long as possible and safeguard the quality of life of patients, their family members, and caregivers through participation and engagement in stimulation, help, and support activities (Flynn and Roach, 2014; Hu et al., 2018).

Non-pharmacological approaches include therapy for patients and their families, associations for patients’ relatives, day care centers, and various economic subsidies, all aimed at providing comprehensive care for patients while mitigating the physical and psychological fatigue associated with caring for a person with dementia (Patel et al., 2014; Casey et al., 2017). Tom Kitwood (Kitwood, 1997) developed the concept of “*person-centered care for people with dementia*” based on the idea that besides neurological damage, dementia treatment should also target aspects such as the personality, history, health, and social environment of each person. Studies conducted using this model in patients with Alzheimer-type dementia (ATD) have found that it helps maintain quality of life for a longer period of time (Sjögren et al., 2012; Yakimicki et al., 2018).

This approach includes animal-assisted therapy, defined as “*an intervention in which an animal is incorporated as an integral part of the treatment process, in order to promote an improvement in physical, psychosocial, and/or cognitive functioning of the person treated*” (Delta Society, 1992). Other studies have used technological elements such as robots with an animal appearance, since some patients and workers may be scared of, allergic to, or averse to animals, which would hinder implementation of such therapies (Valenti Soler et al., 2015; Moyle et al., 2016).

The few studies that have used dogs with subjects with dementia have obtained positive results as regards quality of life, finding an improvement in physical, behavioral, and psychological symptoms (Sellers, 2005; Moretti et al., 2011; Nordgren and Engström, 2012, 2014; Olsen et al., 2016; Tournier et al., 2017). However, most studies in the literature reviewed were conducted with patients with mild to moderate dementia, and there is little information available on patients with severe dementia.

In line with the person-centered care model, which values all people as individuals with a unique history, is committed to promoting their independence, and recognizes the influence of their social environment and the importance of support available to them (McCormack, 2004; Nolan et al., 2004), the aim of the present study was to determine whether canine-assisted therapy maintained or changed the quality of life of people with severe ATD over time. And the hypothesis “The intervention program in Dogs Assisted Therapy (TAP), increases the quality of life of people with dementia.”

## Materials and Methods

### Participants

Participants comprised 10 subjects with ATD, with a mean age of 77.6 years (SD = 9.24). Of these, six were women (mean age 78.8; SD = 10.79) and four were men (mean age 75.8; SD = 7.36).

Our inclusion criteria were as follows: (a) having a diagnosis of Alzheimer’s made by a neurology specialist; (b) meeting the NINCDS-ADRDA criteria for probable Alzheimer’s (McKhann et al., 2011); (c) meeting the DSM-IV criteria for dementia (American Psichiatric Association, 2002); (d) degree of severity between 6 and 7 according to Reisberg’s Global Deterioration Scale (GDS) (Reisberg et al., 1982); (e) not being allergic to dogs, confirmed by means of the clinical history; (f) not being scared of dogs; (g) being a regular user of the day care center run by the Association of Relatives of People with Alzheimer’s and Other Dementias, which employs the person-centered care model; and (h) having given informed consent signed by the patient’s relatives or guardians.

Six of the participants presented a GDS score of 6 (severe cognitive decline) and four a GDS score of 7 (very severe cognitive decline).

### Measurement Instruments

We used Reisberg’s *Global Deterioration Scale* (Reisberg et al., 1982) to assess the severity of cognitive decline.

Quality of life was measured using the *Quality of Life in Late-Stage Dementia* (QUALID) scale developed by Weiner et al. (2000) and adapted for the Spanish population in 2010 (Garre-Olmo et al., 2010). Data for the Spanish population present acceptable internal consistency, with a Cronbach’s coefficient of 0.72, a between-subject reliability of 0.739, and a between-assessor reliability of 0.945. This validation was carried out with people with advanced dementia (mean MMSE: 4) resident in various nursing homes. The QUALID scale consists of 11 items that refer to observable behaviors indicative of the individual’s experience in relation to quality of life (QoL). These include observation of patients’ affective and subjective states in their daily life (smiles, cries, appears sad, upset, irritable, or calm) and behaviors indicating comfort or discomfort in basic activities of daily living considered important from a social point of view (enjoys eating, enjoys touching or interacting with others). The items are scored according to frequency of occurrence on a 5-point Likert scale. The total score for the scale ranges from 11 (best QoL) to 55 (worst QoL).

### Procedure

The intervention was implemented in weekly individual sessions in the morning that lasted half an hour and continued for 12 months. The dog used for the therapy was a Labrador that had been specifically trained for this purpose by a canine specialist. It had been socialized, had a stable, friendly nature, was clinically healthy, and had been properly vaccinated against infectious diseases (rabies, parvovirus, distemper, hepatitis, and leptospirosis). The intervention was carried out by a professional trained in canine-assisted therapy.

It consisted of a skills program that included guided motion exercises aimed at achieving good personal hygiene, maintaining or improving balance, and training and improving movement and walking (voluntary initiation of walking, running in a straight line, turning around, and stopping voluntarily), together with animal-human interaction techniques aimed at improving affective state, mood and anxiety control and reducing inactivity (eye-tracking, stroking, and reacting to stimuli from the dog).

Data were collected at three different times: before starting therapy and at 6 and 12 months after starting, and in all cases were collected by the same professional, an expert in conducting assessments of this type. For the first assessment, observable data were collected prior to starting animal-assisted therapy. At 6 and 12 months, data were collected during the course of the half hour session.

### Statistical Analysis

Data were collected and tabulated using Microsoft Excel®and subsequently exported to the statistical program SPSS v 24.0 for analysis.

Due to on the sample characteristics, we used Friedman’s non-parametric test and the Wilcoxon signed-rank test to determine differences in quality of life between the three data collection times.

### Ethical and Legal Considerations

This study adhered to the ethical standards established by the committee of institutional and/or national research, the Helsinki Declaration of 1964, its subsequent amendments, and comparable ethical standards (Declaración de Helsinki de la Asociación, 1964).

Given that the participants presented severe cognitive decline, informed consent was signed by their families. The form described the study goals and procedures, explained that participation in the study was entirely voluntary and that participants could withdraw at any time without explanation, that the study did not involve any physical risks to participants, and that these would not receive any economic remuneration for participating, and guaranteed that data confidentiality would be maintained in line with the legal requirements set out in the Law on the Protection of Personal Data (Cronbach, 2017).

The study was reviewed and approved by the Ethical Committee of the Association of Relatives of People with Alzheimer’s Disease and other Dementias of León.

### Ethics Committee Data

Ethics Committee of Association of Relatives of people affected by Alzheimer’s disease or other dementias of León – Alzheimer León; C/Photographer Pepe Gracis s/n, 24005, León (Spain). info@alzheimerleon.org. Tel.: 987 26 07 96. C.I.F.: B-24200081. The consent procedure was approved by the Ethics Committee that approved the study.

## Results

**FIGURE 1:**
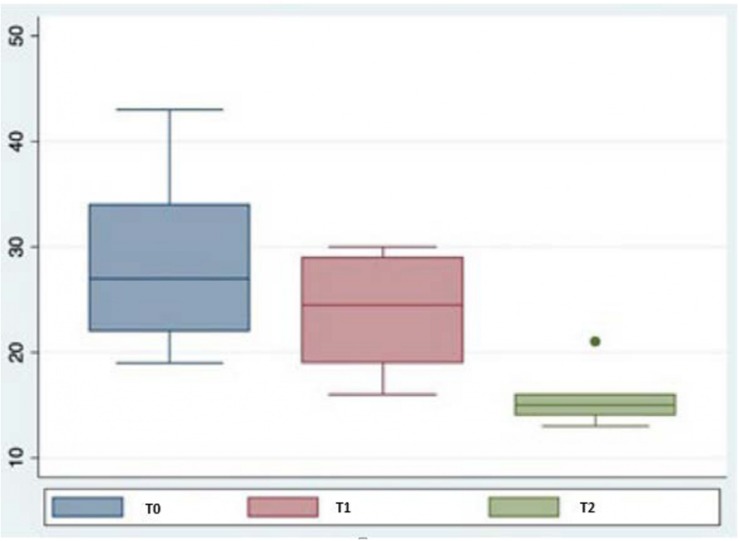
Box plot. Total scores obtained for QUALID.

Figure 1 presents a box plot of total QUALID scale scores for the three different assessment times (before therapy and at 6 and 12 months after starting). As can be seen, scores after the canine-assisted therapy intervention were lower and less dispersed, although there was an outlier in the assessment performed at 12 months, corresponding to a value of 21.

Table 1 shows the results obtained in the Fiedman and Wilcoxon signed-rank tests, observing that the total score on the QUALID scale increases significantly at 6 months and the year of initiation of dog-assisted therapy.

**TABLE 1:** Median scores for QUALID and non-parametric tests.

	**M (SD)**			**Friedman**	**Wilcoxon ranks**		
**QUALID**	**T0**	**T1**	**T2**		**T0–T1**	**T0–T2**	**T1–T2**
Smiles	2.9 (1.66)	2.6 (1.58)	1.8 (1.14)	χ^2^ = 3.77 *p* = 0.152	*Z* = −0.97^*^ *p* = 0.332	*Z* = −1.62^*^ *p* = 0.105	*Z* = −1.08^*^ *p* = 0.279
Appears sad	2.90 (1.73)	2.1 (1.2)	1.1 (0.31)	χ^2^ = 12 *p* = 0.002	*Z* = −2.27^*^ *p* = 0.023	*Z* = −2.26^*^ *p* = 0.024	*Z* = −2.23^*^ *p* = 0.026
Cries	1.9 (1.66)	1.2 (0.63)	1.1 (0.31)	χ^2^ = 4.91 *p* = 0.086	*Z* = −1.60^*^ *p* = 0.109	*Z* = −1.60^*^ *p* = 0.109	*Z* = −0.44^*^ *p* = 0.655
Has facial expression of discomfort	2.3 (1.16)	1.6 (0.69)	1 (0.00)	χ^2^ = 12.56 *p* = 0.002	*Z* = −2.33^*^ *p* = 0.02	*Z* = −2.39^*^ *p* = 0.017	*Z* = −2.12^*^ *p* = 0.034
Appears physically uncomfortable	2.4 (1.51)	1.8 (1.13)	1.3 (0.67)	χ^2^ = 7.44 *p* = 0.024	*Z* = −1.86^*^ *p* = 0.063	*Z* = −2.07^*^ *p* = 0.038	*Z* = −1.29^*^ *p* = 0.197
Verbalizations suggest discomfort	2.6 (1.51)	1.9 (1.10)	1.6 (1.35)	χ^2^ = 3 *p* = 0.223	*Z* = −1.67^*^ *p* = 0.096	*Z* = −1.55^*^ *p* = 0.121	*Z* = −0.75^*^ *p* = 0.453
Is irritable or aggressive	2.3 (1.57)	1.8 (1.03)	1.4 (1.26)	χ^2^ = 6.33 *p* = 0.042	*Z* = −1.89^*^ *p* = 0.059	*Z* = −1.84^*^ *p* = 0.066	*Z* = −1.41^*^ *p* = 0.157
Enjoys eating	2.7 (1.77)	2.9 (1.66)	1.4 (1.27)	χ^2^ = 10.33 *p* = 0.006	*Z* = −1.00^*^ *p* = 0.317	*Z* = −2.04^*^ *p* = 0.041	*Z* = −2.23^*^ *p* = 0.026
Enjoys touching/being touched	3.1 (0.88)	2.7 (0.82)	2.1 (0.88)	χ^2^ = 9.58 *p* = 0.008	*Z* = −1.63^*^ *p* = 0.102	*Z* = −2.26^*^ *p* = 0.024	*Z* = −1.86^*^ *p* = 0.063
Enjoys interacting with others	3.1 (0.57)	2.7 (0.48)	2 (1.15)	χ^2^ = 6.35 *p* = 0.042	*Z* = −1.63^*^ *p* = 0.102	*Z* = −2.15^*^ *p* = 0.031	*Z* = −1.82^*^ *p* = 0.068
Appears calm and comfortable	2.5 (1.43)	1.9 (0.88)	1 (0.0)	χ^2^ = 11.57 *p* = 0.003	*Z* = −2.12^*^ *p* = 0.034	*Z* = −2.26^*^ *p* = 0.024	*Z* = −2.25^*^ *p* = 0.024
Total	28.70 (7.68)	23.80 (4.78)	15.80 (2.86)	χ^2^ = 19.54 *p* = 0.000	*Z* = −2.67^*^ *p* = 0.008	*Z* = −2.81^*^ *p* = 0.005	*Z* = −2.81^*^ *p* = 0.005

^*^Based on positive rankings.

In the item analysis we can verify that the mean and the standard deviation are decreasing as the months of therapy pass, confirming that the increase is statistically significant in the items: “Apears sad,” “Has facial expression of discomfort,” “Appears physically uncomfortable,” “Ocular contact with external stimuli,” “Communicative intention with the stimulus,” “Communicative intention with the professional,” “Connects with the environment and focuses attention,” “Maintains attention with the stimulus during the sessions,” “Interest in activities,” “Emotional connection with the reference professional,” “Enjoys eating,” “Enjoys touching/being touched,” “Enjoys interacting with others,” and “Apears calm and comfortable” (Table 1).

## Discussion

The aim of this study of person-centered care for people with dementia was to determine whether canine-assisted therapy maintained or changed the quality of life of people with severe ATD over time (1 year). The dimensions that the present study wanted to take into account, as far as quality of life is concerned, are those that can be measured through observable behaviors about the patient’s activity and the state of their emotional, both circumstances marked by the measuring instrument used: Quality of Life in Late-Stage Dementia (QUALID). This scale concretely measures the quality of life related to health: what has been tried therefore in the present study is to measure the impact that the disease, and the consequent treatment, have on the perception of satisfaction and on the physical and psychic wellbeing the observer has evidenced through the scale through observation.

An individual analysis of each scale item indicated that scores fell over the course of therapy. These items, including smiles, appears sad, cries, has a facial expression of discomfort, appears physically uncomfortable, makes noises that suggest discomfort, or is irritable, indicated that therapy improved participants’ state of comfort.

This suggests that animal-assisted therapy exerts a positive effect on recipients. Our results are consistent with those reported in previous studies using other animals, including horses (Boletín Oficial del Estado, 1999; Lima and de Sousa, 2004; Dimitrijević, 2009; Oropesa Roblejo et al., 2009), dolphins (Lima and de Sousa, 2004; Antonioli and Reveley, 2005; Oropesa Roblejo et al., 2009) and cats (Stasi et al., 2004; Torres Martínez, 2006; Oropesa Roblejo et al., 2009), which obtained beneficial effects on physical, behavioral, and psychological symptoms, resulting in an improved quality of life.

The data obtained show a significant reduction in total scores for the QUALID scale, suggesting the importance of using this type of therapy in people with severe ATD, which could have a significant impact on caregivers and family members, as noted in previous studies (Gómez García et al., 2017; Lucero Mejicano and González-Ramírez, 2017). In addition, they have important clinical implications for professionals who work with these types of patients. Due to the absence of curative therapies for Alzheimer’s disease, one of the objectives of the care of patients with AD is to preserve their quality of life, which leads us to suggest that it would be appropriate to implement this type of therapy from the initial phase within of integrated care for patients with DTA. As it has been shown in the scientific literature, a higher quality of life is associated with lower levels of depression (Bosboom et al., 2012), a decrease in the presence of neuropsychiatric symptoms (Matsui et al., 2006; Gomez-Gallego et al., 2012) and less dependence on activities of daily life (Nakanishi et al., 2011; Conde-Sala et al., 2013). The clinical impact of this study is directly related to the fact that this type of therapy is not going to have an impact only on the person diagnosed with Alzheimer’s in its final phase, but also has an impact on the direct caregiver and the family nucleus in general, because we can’t ignore that people suffering from dementia in this stage require others to perform their basic activities of daily life. Dementia does not have any treatment for its cure and that is why this study has investigated alternatives, which help both these people and their environment and on a larger scale, to society, to carry in the best possible way the consequences generated by this disease.

The dispersion observed in the scores at the three different assessment times (before therapy and at 6 and 12 months after starting) may have been due to the characteristics of the scale used, among other factors. Measuring the quality of life of people with ATD presents difficulties due to the multidimensionality and subjectivity of the concept (Garzón-Mardonado et al., 2017).

One novelty of the present study with respect to previous research was that data were exhaustively collected over the course of 1 year: in the literature reviewed, no studies were found that exceeded 6 months’ duration (Sellers, 2005; Moretti et al., 2011; Nordgren and Engström, 2012, 2014; Valenti Soler et al., 2015; Olsen et al., 2016; Tournier et al., 2017). Participants were assessed by the same person at each assessment time, yielding a more objective data collection procedure by eliminating any possible discrepancies in assessment when carried out by different professionals.

Caution should be exercised when extrapolating our findings, because the study was conducted with a small sample (*n* = 10) and only included patients with a GDS score of 6 and 7. In future research, it would be useful to increase the number of participants and include patients with mild to moderate degrees of severity.

Our findings provide important information about the benefits for quality of life in patients with ATD of canine-assisted therapy as a complement to comprehensive treatment.

## Data Availability

All datasets generated for this study are included in the manuscript and/or the supplementary files.

## Ethics Statement

They have been cared for and treated in accordance with the guidelines of Alzheimer León.

## Author Contributions

Conceptualization: LS-V, EF-M, and VL; methodology: LS-V, EF-M, and SL-R; software: LS-V and SL-R; validation: LS-V and SL-R; formal analysis: LS-V, EF-M, and SL-R; investigation: LS-V, EF-M, SL-R, AL-A, ED, and VL; resources: LS-V and SL-R; data curation: LS-V and SL-R; writing the original draft: LS-V, EF-M, and VL; preparation: LS-V; writing-review and editing: LS-V, SL-R, ED, AL-A, ED, and VL; visualization: LS-V; supervision: LS-V; project administration: LS-V and EF-M; funding acquisition: LS-V and EF-M.

## Conflict of Interest Statement

The authors declare that the research was conducted in the absence of any commercial or financial relationships that could be construed as a potential conflict of interest.
